# Effect of Formulation on the Binding Efficiency and Selectivity of Precipitation Molecularly Imprinted Polymers

**DOI:** 10.3390/molecules23112996

**Published:** 2018-11-16

**Authors:** K. Fremielle Lim, Clovia I. Holdsworth

**Affiliations:** Discipline of Chemistry, School of Environmental and Life Sciences, University of Newcastle, Callaghan 2308, NSW, Australia; kostafremielle.lim@uon.edu.au

**Keywords:** molecularly imprinted polymers, precipitation polymerisation, molecularly imprinted microspheres, theophylline imprinted polymers, caffeine imprinted polymers, MIP formulation quantitative NMR

## Abstract

This study investigated the effect of feed formulation: the template:functional monomer (T:fM) and functional monomer:crosslinker (fM:X) ratios as well as the initiator concentration, on the binding performance and selectivity of caffeine (CAF) and theophylline (THP) imprinted polymers obtained by precipitation polymerisation in acetonitrile at 60 °C using methacrylic acid (MAA) and ethylene glycol dimethacrylate (EGDMA) as functional monomer and crosslinker, respectively. Template incorporation, monitored by quantitative ^1^H-NMR spectroscopy, ranged from 8 to 77% and was found to be more favourable at both high and low T:fM ratios, low fM:X ratio and high initiator concentration. The resulting T:fM ratio in most MIPs were found to be lower than their feed ratios. Incorporation of THP into the polymers was observed to be consistently higher than CAF and, for most MIPs, the observed binding capacities represent less than 10% of the incorporated template. Improved imprinting factors were obtained from molecularly imprinted polymers (MIPs) with high crosslinker content, i.e., fM:X ratio of 1:10, and high initiator concentration, i.e., initiator:total monomer (I:tM) ratio of 1:5, while T:fM ratio (1:2 to 1:8) was found not to influence binding capacities and imprinting factors (IF). The NIPs showed no preference for either CAF or THP in competitive selectivity studies while MIPs were observed to bind preferentially to their template with THP displaying higher selectivity (72–94%) than CAF (63–84%). Template selectivity was observed to increase with increasing initiator concentration, with MIPs from I:tM ratio of 1:5 shown to be the most selective towards CAF (84%) and THP (93%). The fM:X ratio only showed minimal effect on MIP selectivity. Overall, for the MIP systems under study, template incorporation, binding capacity, imprinting factor and selectivity are enhanced at a faster rate of polymerisation using an I:tM ratio of 1:5. Polymer particles obtained were between 66 to 140 nm, with MIPs generally smaller than their NIP counterparts, and have been observed to decrease with increasing T:fM and fM:X ratios and increase with increasing initiator concentration.

## 1. Introduction

Molecularly imprinted polymers or MIPs are synthetic receptors with pre-determined molecular recognition capabilities specific for its target molecule [1,2,3,4]. Widespread applications of these materials are due to their selectivity and specificity which are comparable with biomolecules (e.g., enzymes and antibodies) but without the associated issues [5,6,7,8]. MIPs are more economical to use than biomolecules since they can be reused and have longer degradation times. Due to these advantages, MIPs have been widely used in more applications than biomolecules, and are continuously being studied for more applications, such as sensors, separation chemistry, and chromatography [6,9,10,11,12,13].

The process of non-covalent molecular imprinting requires a template, usually the target molecule or an analogue, a functional monomer, a crosslinker and a porogenic solvent. The template molecule, by virtue of its functional groups or association sites, forms a loosely associated pre-polymerisation cluster with the functional monomer. Subsequently, this cluster is joined together, in a three-dimensional framework, by copolymerisation of the functional monomer with the crosslinker usually by radical polymerisation. The template is then removed from the imprinted polymers, leaving a cavity that is complimentary in shape and functionality with the template. 

A number of previous studies focused on investigating the extent of influence of the feed formulation on the binding performance of monolithic MIPs have yielded contradicting results. For instance, in terms of the effects of the ratio of functional monomer to the cross-linker (fM:X), separate studies by Yilmaz [14] and Tom [15] reported a decrease in the efficiency of theophylline and sulfadimethoxine MIPs, respectively, with increasing fM:X feed ratio (fM:X of 1:2–1:4 [14] and fM:X of 1:1–1:5 [15]). Yilmaz et al. illustrated that while the MIP monoliths produced at higher fM:X ratios have comparable recognition capabilities with those prepared at lower fM:X ratios, they have lower number of apparent high quality binding sites (as their NIPs also showed higher template binding), resulting in lower imprinting factor (IF) values. The authors have attributed this observation to the optimum rigidity of the polymers: higher fM:X (lower concentration of X) produced polymers with weak and less rigid cavities that are unable to trap and retain the template during the imprinting process. Sellergren [16] and Golker [17], on the other hand, showed that higher fM:X feed ratios (fM:X = 1:1 to 1:4) produced more binding efficient MIP monoliths with higher IFs which was explained by Golker et al. to be due to the effects of the increase in the hydrogen bond interaction between the template and the functional monomer according to their molecular simulations study. In the case of Sellergren et al.’s study, the monolithic L-phenylalanine anilide MIPs were also found to be more enantioselective. Golker et al. further illustrated that higher fM:X feed ratios yielded bupivacaine MIP monoliths with bigger pore sizes. 

Similarly, published results focused on the effect of template:functional monomer (T:fM) feed ratio on the binding efficiency of the monoliths have been inconsistent. Andersson et al. and Tom et al. illustrated that lower T:fM feed ratios (T:fM = 1:6 vs. T:fM = 1:4) resulted in higher binding efficient nicotine [18] and sulfadimethoxine [15] MIPs, respectively, attributed to the formation of higher number of homogenous binding sites in monoliths at lower T:fM ratios [19]. Rampey et al., on the other hand, demonstrated that increasing the concentration of template in the feed (i.e., higher T:fM feed ratio), increases the number of binding sites, N and ultimately the IF [20]. This is consistent with the results published by Andersson et al. [19] which suggested that the concentration of fM in the feed should be kept at a minimum amount to reduce non-specific analyte binding.

While bulk molecular imprinting has been commonly used [21,22,23], only a few studies have investigated the correlation of feed formulation to the binding performance of molecularly imprinted microspheres prepared by precipitation polymerisation. Yoshimatsu et al. found the binding efficiency of the (*R*,*S*)-propanolol microspheres to be greatly affected by the fM:X ratio (varied from 1:1 to 1:4) in the feed. Higher concentration of MAA resulted in lower binding capacity (~2 times lower or halved) attributed to the loss of rigidity and selectivity of the polymers [24]. Similarly, lowering the amount of MAA, to MAA:TRIM = 1:2.3, also gave a similar effect attributed to insufficient concentration of the T-fM complex formed during association stage [24]. Similar results were obtained by Xia et al. [25] who observed a decrease in the number of binding sites in the kaempferol imprinted microspheres with increasing ratios of MAA:EGDMA from 1:2 to 2:1. Both groups have concluded that there should be a balance between the concentration of cross-linker, which provides the rigidity of the microspheres, and the functional monomer, which dictates the amount of T:fM complexes formed, to be able to optimise the fM:X feed ratio for a particular system [26]. The most binding efficient MIP microspheres according to published results are the polymers synthesized in fM:X between 1:4–1:6 [25]. 

In this study, we investigated the effect of feed formulation, particularly the functional monomer:cross-linker (fM:X) and template:functional monomer (T:fM) ratios, on the binding performance of caffeine (CAF) and theophylline (THP) imprinted microspheres obtained by precipitation polymerization in acetonitrile at 60 °C using methacrylic acid (MAA) and ethylene glycol dimethacrylate (EGDMA) as fM and X, respectively. In addition, we also examined the effect of the concentration of the initiator as, to date, there has only been limited reports [27,28,29] on its effect on imprinting and binding efficiencies. A number of papers have alluded to significant size difference between the non-imprinted and imprinted particles obtained by precipitation polymerisation [24,30,31,32,33,34,35,36,37,38] which imposes difficulty in expressing the actual efficiency of the MIP particles as it is current practice to compare it with its non-imprinted version. Thus, we also report our effort to correlate particle size to feed formulation and polymer composition. 

**Figure 1:**
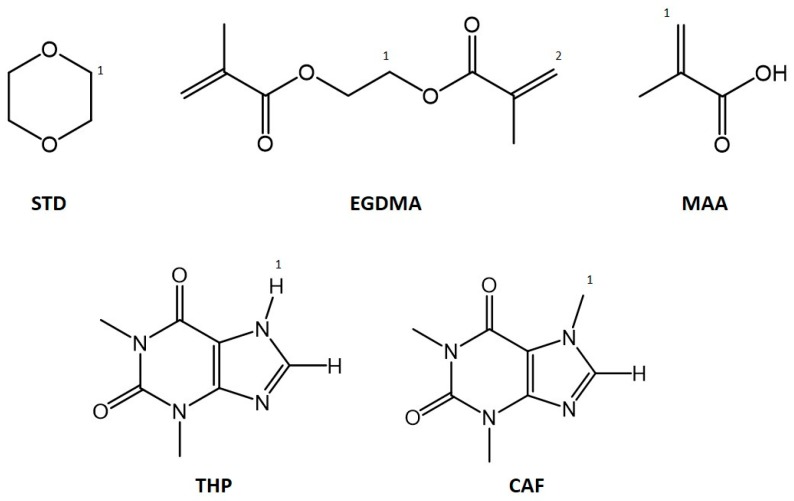
Structures of 1,4-dioxane (**STD**), ethylene glycol dimethacrylate (**EGDMA**), methacrylic acid (**MAA**), theophylline (**THP**) and caffeine (**CAF**). Labelled atoms correspond to nuclei used for NMR analysis.

An added value to this study is the application of quantitative ^1^H-NMR spectroscopy (q-NMR) in monitoring template incorporation and determination of the composition of the imprinted microspheres which have not been addressed by previous studies. Employing 1,4-dioxane as an external standard (**STD**, Figure 1), the unreacted components (EGDMA, MAA and the template) left in the post-polymerisation solution were calculated and compared with the amounts in the pre-polymerisation solution with the assumption that the difference was incorporated in the polymer (see Section 3.3). 

Template incorporation ranging from 8 to 77% was found to be more favourable at both high and low T:fM ratios, low fM:X ratio and high initiator concentration. The resulting T:fM ratio in most MIPs were found to be lower than their initial feed ratios. Incorporation of THP into the polymers was consistently higher than CAF and, for most MIPs, the observed binding capacities were less than 10% of the incorporated template. We have attempted to correlate template incorporation and rebinding but it is worth noting that this comparison is difficult to interpret as the latter is dependent on rebinding conditions. 

The results obtained in varying the polymerisation feed suggest that T:fM ratios between 1:2 to 1:8 give comparable binding capacities regardless of the level of template incorporation. Higher binding performance was obtained from MIPs of higher crosslinker content, i.e., fM:X ratio of 1:10, confirming the importance of polymer rigidity in producing binding efficient polymers. It has also been demonstrated that faster polymerisation reaction due to high initiator concentration for the systems under study, i.e., I:tM ratio of 1:5, is more favoured. 

Results of the selectivity assays between CAF and THP showed NIPs to have no preference for either CAF or THP while MIPs were observed to bind preferentially to their template, with THP displaying higher selectivity than CAF. While the fM:X ratio only showed minimal effect, MIP selectivity has been observed to markedly increase with increasing initiator concentration with MIPs prepared from I:tM ratio of 1:5 showing template selectivity as high as 84% and 93% for CAF and THP, respectively.

## 2. Results and Discussion

Three different experiments which involve varying the ratios of different components in the polymerisation feed: initiator:total monomer (I:tM), functional monomer:cross-linker (fM:X) and template:functional monomer (T:fM), were performed and assessed (see ESI Table S1) using xanthine derivatives caffeine (CAF) and theophylline (THP) (Figure 1) as templates. THP is both a hydrogen bonding donor and acceptor while CAF is only a hydrogen bonding acceptor [39]. Consequently, previous studies have demonstrated more favourable interaction, i.e., higher binding energy, between THP with functional monomer MAA [40] compared to MAA and CAF [41] as was calculated by molecular modelling. Both CAF and THP are two of the commonly used templates in molecular imprinting with high association constants: between 1.62–5.43 × 10^3^ M^−1^ for CAF [42,43] and between 1.53 × 10^4^ to 1.0 × 10^8^ [14,44] for THP. 

### 2.1. Variation of Template to Functional Monomer (T:fM) Ratios

MIPs of CAF and THP were synthesized from various T:fM ratios: 1:2 (TM2), 1:6 (TM6) and 1:8 (TM8) and compared to the MIP prepared using the most commonly used T:fM of 1:4 (TM4) [45]. Polymers were thermally synthesized in an oil bath at 60 °C using AIBN as the initiator, MAA as the functional monomer, EGDMA as the cross-linker and ACN as porogen employing the most commonly used and accepted fM:X ratio of 1:5 [45]. The formulation was based upon previously reported studies on CAF and THP MIPs, i.e., I:tM of 1:100 [46] and 10 mL of porogen per mmol of the total monomer (mmol EGDMA + mmol MAA) [47]. This porogen/monomer ratio is within the range that has been reported for precipitation polymerisation, e.g., 4 mL/mmol [48], 12 mL/mmol [49] and 16 mL/mmol [9,50]. 

The unreacted components (EGDMA, MAA and the template) left in the post-polymerisation solution were calculated by q-NMR and compared with the amounts in the pre-polymerisation solution with the assumption that the difference was incorporated in the polymer. All CAF and THP MIPs and their corresponding NIPs prepared from varying T:fM ratios incorporated between 83–93% (with respect to the initial amount) of EGDMA (Table S1). Conversely, the polymers also showed minimal differences in the amount of incorporated MAA from 75–88% for the NIPs and 74–87% for the MIPs. These resulted in fM:X ratios of 1:5–6, very close to the initial fM:X ratio of 1:5, and suggest that the concentration of template in the feed has minimal effects on the composition of the resulting polymers. Polymer yields, estimated from NMR, were in the range of 83–91% for the NIPs and 84–93% for the two MIPs.

**Figure 2:**
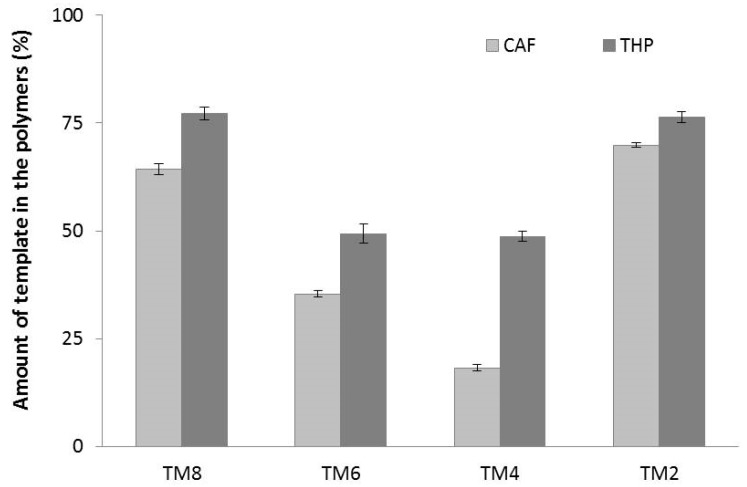
Percentages of CAF and THP incorporated in the polymers with respect to the feed at various T:fM ratios measured by ^1^H q-NMR using 1,4-dioxane in DMSO-*d*_6_ as the reference standard.

Figure 2 shows that template incorporation, expressed as percentage with respect to the initial concentration of template in the feed, varies from a low of 18 ± 1% to a high of 77 ± 1% favouring high template (TM2) and high functional monomer (TM8) concentrations. In all cases, THP MIPs incorporated higher amounts of template compared to CAF polymers indicating a stronger interaction between MAA and THP than with CAF. The gradual decrease in template incorporation in the polymers with decreasing concentration of MAA in the feed, from TM8 to TM4, is consistent with Le Chatelier’s principle. At high MAA concentration (TM8), the formation of T-fM complexes is highly favoured thereby incorporating more template. Conversely, high template concentration (TM2) favours the formation of the T-fM complexes (which may not necessarily be of optimum stochiometries) also in accordance with Le Chatelier’s principle. The T:fM feed ratio of 1:2 was observed to be preserved for both the TM2_THP_ and TM2_CAF_ polymers but lower T:fM ratios in the polymers (between 1:10–1:18) were obtained for the other MIPs in the series (Table S1). Both TM2_THP_ (70 nm) and TM2_CAF_ (66 nm) were also significantly smaller (by 10–70 nm, Table S2) than the other polymers suggesting that the template could have acted as nucleation points during the polymerisation, pulling the monomer inwards, and producing particles with smaller *d*_H_ in accordance with the results of Yoshimatsu [30]. The differences in the particle sizes are also evident in the SEM images shown in Figure S1.

**Figure 3:**
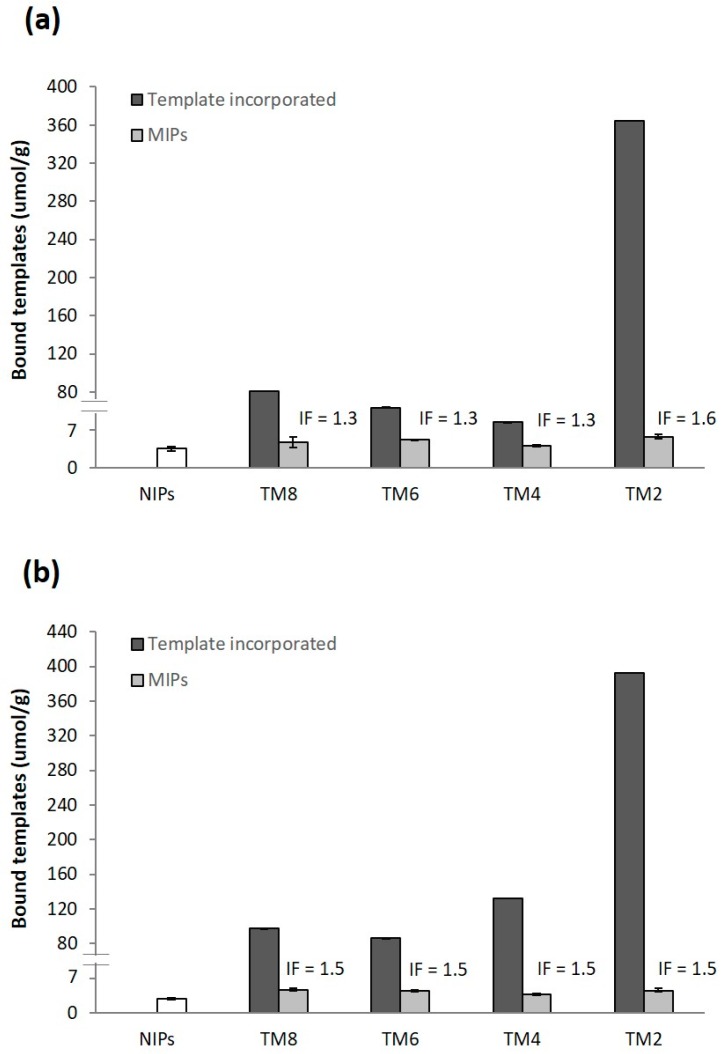
Template incorporation and binding of (**a**) CAF and (**b**) THP MIPs and NIPs synthesized at various T:fM ratios. Polymers were incubated for 18 h in 100 μM template rebinding solution with 70–90% binding from the rebinding solution. Post rebinding solutions were analysed by HPLC. Template incorporation in µmol/g is also shown for comparison purposes.

Following a previously reported protocol [51], 10.0 mg of the polymers were incubated for 18 h in 1 mL of 100 µM of CAF or THP in acetonitrile and were observed to bind 70–90% of the template. The binding results, summarised in Figure 3, showed MIP binding to be higher than NIP binding (4.1 ± 0.4 µmol/g) for all T:fM ratios indicating successful formation of imprints. In the case of CAF MIPs (Figure 3a), minimal difference in binding was observed, with TM2_CAF_ rebinding the most (6.6 ± 0.4 μmol/g) and TM4_CAF_ the least (4.7 ± 0.2 μmol/g), resulting in IFs of 1.3 to 1.6. Figure 3 also shows a comparison between the amount of rebound and incorporated template. TM4_CAF_, exhibiting the lowest template incorporation among the TM_CAF_ series, recorded the highest conversion of incorporated to bound sites (referred hereto as the binding site conversion) of 9.5%. TM2_CAF_, on the other hand, only gave a 1.8% binding site conversion despite very high template incorporation. The low binding site conversion observed with TM2_CAF_ could be attributed to the formation of T:fM complexes of less favourable stoichiometries for imprinting due to the presence of higher amounts of CAF in the feed and insufficient concentration of MAA forming incomplete or partial binding sites [52]. These results suggest that there is an optimum number of high fidelity T-fM complexes that could be formed and not necessarily correlated to template incorporation. Kim et al. has also observed that, while the optimum imprinting efficiency of a MIP system is dependent on the T-fM complexes formed during the association process, additional amount of template or functional monomer in the system would not improve the binding efficiency of the polymers [52].

Similar binding results were also obtained for the THP system. The affinity of the NIP towards THP (4.1 ± 0.3 µmol/g) was found to be comparable to that towards CAF as shown in Figure 3b. In the case of MIP binding, no significant difference was observed within the TM_THP_ series, with a high of 6.6 ± 0.7 μmol/g exhibited by TM8_THP_ and a low of 5.4 ± 1.0 µmol/g by TM4_THP_. As with the CAF system, TM2_THP_ also recorded the lowest binding site conversion (1.60%) while the highest binding site conversion was observed for TM6_THP_ (7.45%). The IFs for all TM_THP_ series are comparable (IF = 1.5) suggesting that even with the lowest concentration of template (TM8_THP_), there is already enough amount of template to form the equilibrium concentration of T-fM complexes and that, as with the CAF counterparts, the binding capacity of the MIPs is not dependent on the concentration of the template in the feed, down to T:fM of 1:8 (11% THP).

### 2.2. Variation of Functional Monomer to Cross-Linker Ratio (fM:X) 

Keeping the T:fM ratio constant at 1:4, three sets of CAF and THP MIPs using various fM:X (MAA:EGDMA) ratios: 1:2 (MX2, 33% MAA), 1:5 (MX5, 17% MAA) and 1:10 (MX10, 9% MAA), were prepared. As with the T:fM studies, the I:tM ratio and the porogen volume/total monomer ratio were kept constant at 1:100 and 10.0 mL/mmol, respectively. 

All polymers in the MX series incorporated between 86 and 93% (Table S1) of the EGDMA in the feed, with the highest displayed by MX2 polymers. In terms of MAA, the MX_CAF_ MIPs incorporated between 70% (MX10) and 80% (MX5) while the MX_THP_ polymers showed a wider range of MAA incorporation between 61% (MX10) and 83% (MX2). Both MX2 and MX5 CAF and THP MIPs preserved their initial fM:X ratios of 1:2 and 1:5, respectively. The fM:X ratios in MX10 MIPs, on the other hand, were found to be 1:13 (MX10_CAF_) and 1:14 (MX10_THP_), slightly lower than the initial ratio of 1:10. The incorporation of CAF (Figure S4) was observed to be decreasing with increasing concentration of MAA with MX10_CAF_ recording the highest incorporation of 35 ± 1% and reduced by 3.5 times to 10 ± 1% in MX2_CAF_. Lower incorporation of CAF in high fM:X (higher amount of MAA in the feed) could be attributed to the possibility of MAA preferring to dimerise with itself than interact with CAF thus lowering the chances of forming template-monomer complexes [53,54,55]. The resulting T:fM ratios in the polymers (Refer to Table S1) were observed to be lower, 1:32, 1:18 and 1:8 for MX2_CAF_, MX5_CAF_ and MX10_CAF_, respectively, than the feed ratio of 1:4. In the case of THP (Figure S4), highest THP incorporation was observed in MX5_THP_ (49 ± 2%) while MX2_THP_ and MX10_THP_ incorporated similar amounts (~32%, 1.5 times lower). The increase in THP incorporation from MX10 to MX5, i.e., increasing concentration of MAA, is expected in accordance with Le Chatelier’s principle. However, at fM:X ratio higher than MX5, i.e., MX2, THP incorporation was reduced and was speculated to be due to the competing dimerization of MAA. This effect has been observed to be more prominent with MX_CAF_-MIPs due to weaker T:fM interactions. Similar to the MX_CAF_ MIPs, the resulting T:fM ratios in MX_THP_ MIPs were lower than their feed ratios: 1:11 for MX2_THP_, 1:6 for MX5_THP_ and 1:8 for MX10_THP_. Overall, incorporation of THP in the polymers is higher than CAF undoubtedly due to stronger THP:MAA interactions. 

**Figure 4:**
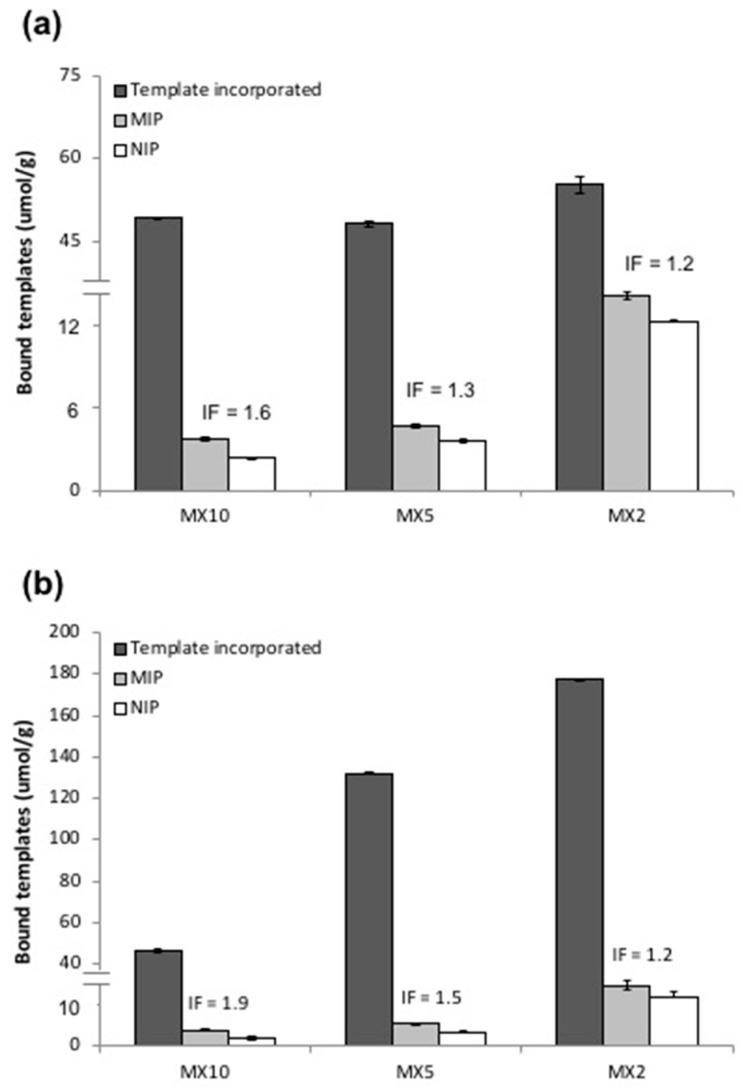
Comparison of the amount of (**a**) CAF and (**b**) THP incorporated and rebound by the MIPs and NIPs prepared from various fM:X ratios. Polymers were incubated for 18 h in 100 μM template rebinding solution and the post rebinding solutions were analysed by HPLC.

As shown in Figure 4a, the amount of bound CAF slightly increased from MX10_NIP_ to MX5_NIP_ (2.4 ± 0.1 μmol/g to 3.6 ± 0.2 μmol/g, respectively) but drastically increased (4×) to 12.0 ± 1.2 μmol/g for MX2_NIP_. This observation is consistent with the results of several studies [24,45,50,51,56,57] indicating the non-specific binding capability of the NIPs to be proportional to the MAA content in the feed. As expected, an increase in the non-specific binding was observed in MX2_NIP_. In terms of MIP binding, MX2_CAF_ was observed to bind the highest, equivalent to 14.1 ± 0.3 µmol/g (25.6% of the incorporated CAF), while MX10_CAF_ bound the lowest with only 3.8 ± 0.1 µmol/g (7.6% of the incorporated CAF). The high binding of MX2_CAF_ is indicative of the formation of higher concentration of T-fM complexes as a result of higher concentration of functional monomer incorporated in the polymer. The lower binding exhibited by MX10_CAF_ polymers is consistent with the published results of Yoshimatsu et al. [24] which, they also attributed to the lower concentration of T-fM complexes formed during the imprinting process [16,24,57,58,59]. Figure 4b also showed that MX2_THP_ incorporated the highest THP (177.3 ± 1.3 µmol/g) for this system, and incorporation of THP decreased by 3.9 times to 45.9 ± 0.1 µmol/g in MX10_THP_ polymers. MX2_THP_ also exhibited the highest MIP binding of 14.7 ± 1.1 μmol/g (8.5% of the incorporated THP) dropping to 4.0 ± 0.2 μmol/g for MX10_THP_ (8.5% of the incorporated THP). Similar to the CAF system, MX2_NIP_ gave the highest THP binding of 12.0 ± 1.2 μmol/g and dropped 3.4× to 3.6 ± 0.2 μmol/g for MX5_NIP_ and 5.7× to 2.1 ± 0.4 μmol/g for MX10_NIP_.

While MX2 MIPs displayed the highest template binding, their corresponding NIPs also showed the highest template binding resulting in low imprinting factors for both MIP systems. On the other hand, despite displaying the lowest template incorporation, MX10 MIPs exhibited the most favourable binding performance, i.e., higher IFs, with their corresponding NIPs showing very low NIP binding. These binding results further confirm the importance of rigidity of the MIPs to their overall performance consistent with the results published by Rosengren et al. [57]. In all cases, a slightly higher MIP binding for MX_THP_ compared to MX_CAF_ polymers was observed, providing higher IFs attributed to a greater interaction between THP and MAA. 

**Figure 5:**
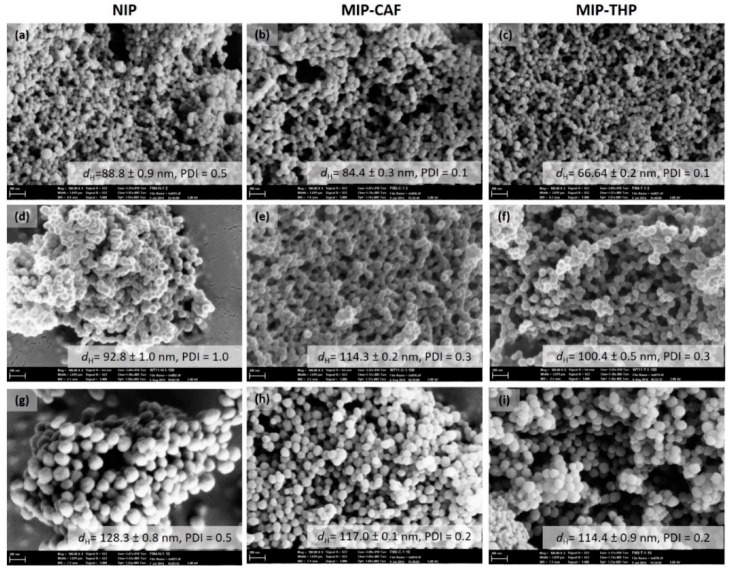
SEM images of polymers synthesized from various fM:X ratios: (**a**) MX2_NIP_, (**b**) MX2_CAF_, (**c**) MX2_THP_, (**d**) MX5_NIP_, (**e**) MX5_CAF_, (**f**) MX5_THP_, (**g**) MX10_NIP_, (**h**) MX10_CAF_ and (**i**) MX10_THP_. Scale bar represents 200 μm at 100,000× magnification. Insets are the hydrodynamic size of the microspheres with the corresponding polydispersity indexes (PDI) measured by DLS.

The DLS measurements showed increasing *d*_H_ with decreasing concentration of MAA in the feed (from MX2 to MX10) which is also evident in the SEM images given in Figure 5. The particle sizes of the NIPs range from 89–128 nm and generally bigger and more polydispersed (PDIs of 0.5–1.0) than their MIP counterparts. MX_CAF_ and MX_THP_ MIPs range from 84–117 nm and 66–115 nm, respectively, with PDIs ranging from 0.1–0.3. Long [60] speculated that the differences in the sizes of the MIPs and the NIPs could be due to the template, by virtue of its interaction, being able to pull the monomers towards it resulting in smaller particles. This inward pull could also explain the difference in size between THP and CAF MIP microspheres, with the former observed to be smaller, quite possibly due to the interaction between THP and the MAA functional monomer being stronger than that with CAF.

### 2.3. Variation of Initiator to Total Monomer (I:tM) Ratio

Piletsky et al. [27,28] have observed that the concentration of initiator affects the rebinding efficiency of bulk MIPs, that is, higher concentration of initiator led to less efficient MIPs which they attributed to be due to the disruption of the formation of a stable T-fM complex due to the release of heat during imprinting process. However, little information is available on its effect on MIP particles prepared by precipitation polymerisation. In our previous study [29], we have showed that template incorporation and rebinding in precipitation MIPs are favoured by a moderate initiator concentration, i.e., initiator:total monomer (I:tM) ratio of 1:131, while low I:tM ratio (i.e., 1:200) drastically reduced template incorporation and binding capacity of the microspheres.

Utilising I:tM ratios of 1:5 (IM5), 1:10 (IM10), 1:100 (IM100), 1:500 (IM500) and 1:1000 (IM1000), polymers were again synthesized thermally (60 °C) using EGDMA as the cross-linker, MAA as the functional monomer and acetonitrile (ACN) as porogen. We opted to use the fM:X ratio of 1:5 for all I:tM experiments as it is the most commonly used fM:X ratio in precipitation polymerization [45]. The T:fM and volume/monomer ratios were kept constant at 1:4 and 10 mL/mmol of the total monomer, respectively. 

Polymer conversions of the IM NIPs were observed to be decreasing with decreasing concentration of initiator consistent with the observation of Stover et al. [61]. Table S1 showed that the EGDMA uptake of the NIPs decreased from 98 ± 1% (IM5_NIP_) to 88 ± 2% (IM500_NIP_) and dropped to 75 ± 3% with IM1000_NIP_ (0.1% initiator). The same behaviour was observed for MAA, with an uptake of 88 ± 2% obtained with IM5_NIP_ and dropping to 39 ± 2% with IM1000_NIP_. Similarly, the degree of crosslinking was also found to decrease with decreasing concentration of initiator from 71% (IM5) to 62% (IM1000). While the polymers with higher concentrations of initiator (IM5_NIP_ and IM100_NIP_) maintained the fM:X feed ratio of 1:5, those prepared with lower concentrations of initiator (IM500_NIP_ and IM1000_NIP_) resulted to fM:X ratios of 1:6 to 1:10. The low fM:X ratios obtained for IM500_NIP_ and IM1000_NIP_ polymers indicate that EGDMA has a higher reactivity than MAA, evident at low concentration of initiator, resulting to a higher concentration of X in the polymers. 

In the case of IM MIPs, the incorporation of EGDMA and MAA in the polymers also decreased with decreasing concentration of initiator in the feed (Table S1). CAF MIPs incorporated between 40–98% EGDMA and 39–92% MAA, while THP MIPs recorded a range of 43–98% EGDMA and 38–93% MAA incorporation. In contrast to the NIPs, however, the initial fM:X ratio of 1:5 was preserved at all I:tM ratios indicating that the presence of the template could increase the incorporation of MAA, particularly at low I:tM ratios, by acting as an efficient nucleation point during polymerization [62,63]. 

**Figure 6:**
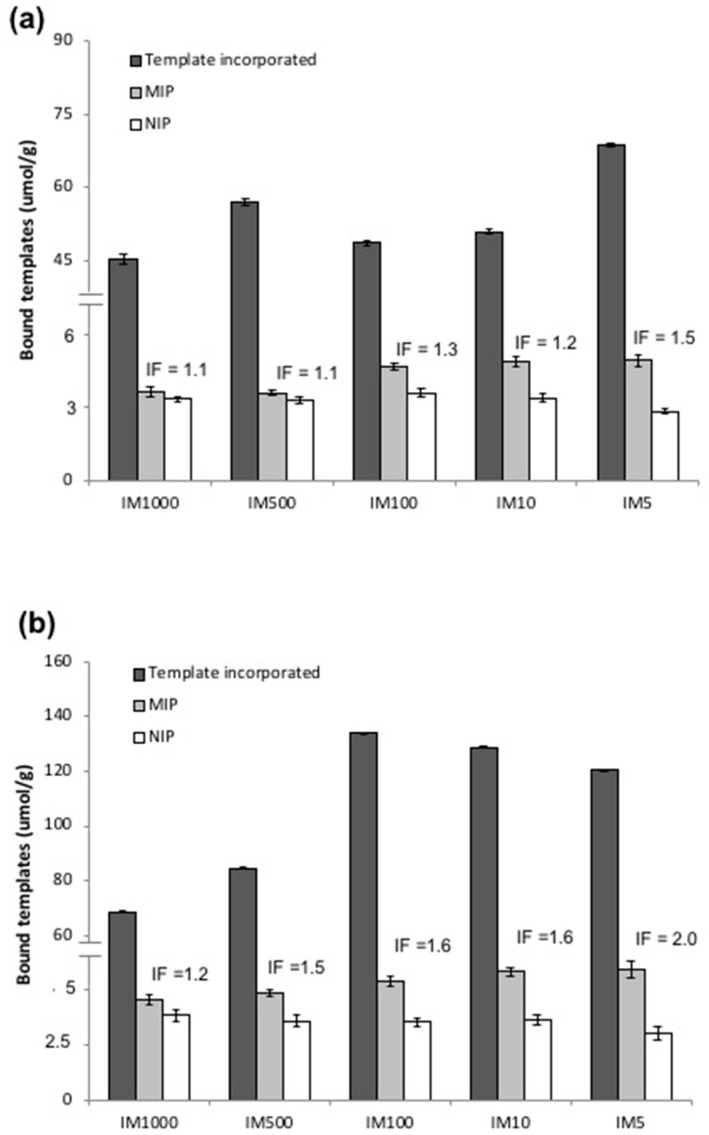
Comparison of the amount of (**a**) CAF and (**b**) THP incorporated and rebound by the MIP and NIP polymers synthesized at various I:tM ratios. Polymers were incubated for 18 h in 100 μM template rebinding solution and the post rebinding solutions were analyzed by HPLC.

Template incorporation (both CAF and THP) was also observed to be decreasing with decreasing concentration of initiator as shown in Figure 6. In the case of the CAF MIPs, IM5_CAF_ recorded the highest template incorporation of 26 ± 1% (69 μmol/g) while IM1000_CAF_ gave the lowest of 8 ± 1% (45 μmol/g). The incorporation of THP is, in all cases, higher than CAF, with the highest value of 50 ± 1% (120 μmol/g, IM5_THP_) almost twice as much as the highest recorded CAF incorporation (26 ± 1%, IM5_CAF_). These resulted to a lower T:fM ratio in the polymers: 1:7 to 1:12 for THP and only 1:14 to 1:20 for CAF. This is another evidence suggesting that the strength of interaction governs the formation of T:fM association clusters. In theory, the rate of reaction in free radical polymerisation is directly proportional to the concentration of the initiator [64,65]. Based on our results, it would seem that template incorporation is favoured by faster polymerisation. This could be due to the “snap-freezing” of the T-fM complexes, put forward by Turner et al. to explain the better performance of the MIPs from microwave induced initiation compared to thermally-synthesized MIPs, allowing the preservation of the three dimensional arrangement leading to the formation of stronger binding cavities in the polymers [43]. Additionally, higher concentration of initiator would result in shorter chain lengths between crosslinks conducive to template entrapment. 

The affinity of the NIPs towards CAF was observed to be increasing with decreasing concentration of initiator (Figure 6a) and could be correlated to the increasing X content within the polymers, but lower degree of crosslinking (see Table S1), providing CAF greater access to polymer surface. In terms of MIP binding, the amount of CAF rebound (4.91 ± 0.24 μmol/g) was observed to be highest for IM5_CAF_ and lowest (3.57 ± 0.71 µmol/g) for IM1000_CAF_. The low binding of IM1000_CAF_ could be attributed to lower template incorporation and polymer conversion due to a slower rate of polymerisation. Considering the low CAF incorporation and relatively high MIP binding of IM1000_CAF_, this polymer recorded the highest binding site conversion of 9.6%, the lowest being 6.2% showed by IM500_CAF_. While MIP CAF binding was observed to increase with increasing amount of initiator, their corresponding NIPs exhibited a reversed trend resulting to IFs ranging from 1.1 to 1.5, with IM5_CAF_ recording the highest IF.

NIP binding towards THP was also observed to increase with the concentration of initiator from 3.07 ± 0.29 µmol/g (IM5_NIP_) to 3.86 ± 0.28 µmol/g (IM1000_NIP_) as shown in Figure 6. In comparison, the MIPs showed the opposite trend with IM5_THP_ binding the highest (5.97 ± 0.36 μmol/g) and IM1000_THP_ the lowest (4.59 ± 0.21 μmol/g). As with the CAF system, the MIP with the highest concentration of initiator, IM5_THP_, recorded the highest IF of 2.0 while IM1000_THP_ gave the lowest IF of 1.2. The highest binding site conversion (6.3%) was recorded by IM1000_THP_ due to a lower incorporation of template while IM5_THP_ gave the lowest of only 5.0% conversion. The low binding site conversion calculated for IM5_THP_ suggests that the high incorporation of THP might be simply due to partial imprinting, not due to the formation of high fidelity binding sites. This could be due to the disruption of the T-fM complexes by the heat given off during the decomposition of high concentration of initiator, as was proposed by Mijangos et al. [27]. Despite the higher binding site conversion of the IM1000 polymers, the use of this IM ratio for the synthesis of MIPs by precipitation is not recommended since, at this initiator concentration, the polymerisation is difficult to control due to its sensitivity to the presence of oxygen. Additionally, IM1000 produced the lowest yield among all of the IM polymers synthesized. Based on IF values and polymer yields, this study showed that a higher concentration of initiator, i.e., I:tM ratio of 1:5, is more preferable to use for precipitation imprinting of CAF and THP under similar conditions.

**Figure 7:**
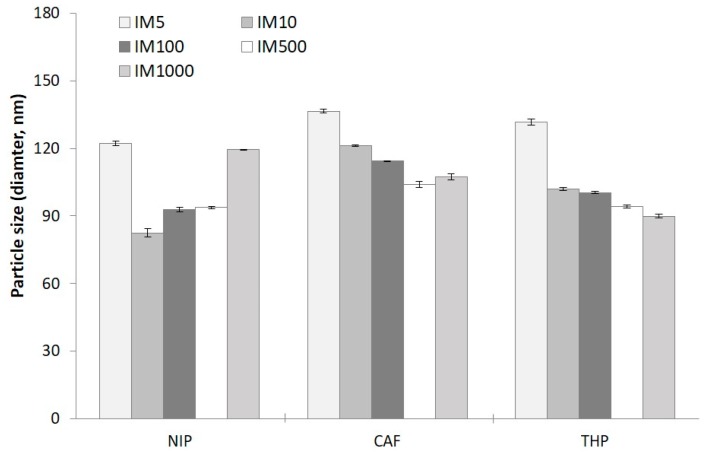
Hydrodynamic sizes, *d*_H_ of microspheres synthesized at various I:tM ratios. Measurements were conducted using Dynamic Light scattering (DLS) and using ACN as the dispersant.

According to published literature, one of the physical properties of the polymers that is evidently affected by the concentration of initiator is the particle size [61,66,67,68]. The DLS-based particle size (*d*_H_) of the NIPs ranges from 83–140 and was observed to follow a bell shape with IM1000_NIP_ giving *d*_H_ comparable to IM5_NIP_ as shown in Figure 7 and Figure S7. At higher concentrations of initiator, more nuclei are formed in a short amount of time which aggregate to give particles of larger diameter [61,66,67,68,69,70]. Quite possibly, even though few nuclei are formed in IM1000_NIP_ due to the low concentration of initiator, the growing polymer nuclei can coalesce and form larger particles. The *d*_H_ of the MIPs, on the other hand, have been observed to increase with increasing concentration of initiator, from 95 to 136 nm for IM_CAF_ and 90 to 132 nm for IM_THP_, consistent with the results shown by several studies [61,66,67]. The THP MIPs are generally smaller than its CAF counterparts and could be attributed to the interaction between the functional monomer MAA and THP being stronger than with CAF. The PDI values of the MIPs are slightly narrower (0.2 to 0.6) than the NIPs (0.3–1.0) but cannot be correlated to I:tM ratios.

### 2.4. Binding Selectivity Studies

The binding selectivities of selected CAF and THP MIPs and NIPs against each other obtained from varying fM:X and I:tM feed ratios were measured after incubating the polymers in equimolar concentrations of CAF and THP. The polymers prepared from various T:fM ratios were not included in the selectivity assays as their binding performance, based on their binding capacities and IFs, did not seem to be affected by the T:fM ratios. The results are summarized in Table 1. It is noteworthy that most NIPs displayed no preference for either CAF or THP with only MX5 and MX10 showing more favourable CAF binding by 16% and 18%, respectively. On the other hand, all MIPs were observed to bind preferentially to their template, with THP selectivity (72–94%) higher than that of CAF (63–84%).

The fM:X ratio does not seem to have a marked effect on MIP selectivity. In the case of CAF MIPs, there is a minimal 8% difference in binding selectivity between the least selective MX2_CAF_ and the most selective MX5_CAF_. Conversely, there is only a 6% difference in binding selectivity between the least selective MX5_THP_ to the most selective MX10_THP_. Interestingly, the MIPs prepared from various I:tM ratios show a 21% increase in binding selectivity to its template with increasing initiator concentration from 63% (IM1000_CAF_) to 84% (IM5_CAF_) for CAF MIPs and from 72% (IM1000_THP_) to 93% (IM5_THP_) for THP MIPs. These results indicate that the formation of high fidelity binding cavities during imprinting is enhanced at higher initiator concentration where the rate of polymerization is faster. 

## 3. Materials and Methods 

### 3.1. Materials and Reagents 

Methacrylic acid (MAA, Sigma Aldrich, Castle Hill, Australia) and ethylene glycol dimethacrylate (EGDMA, Sigma Aldrich, Castle Hill, Australia), were purified by passing through a basic aluminum oxide column. Caffeine (CAF, Sigma Aldrich, Castle Hill, Australia) and theophylline (THP, Sigma Aldrich, Castle Hill, Australia) were used as received. 2,2’-Azobisisobutyronitrile (AIBN, DuPont Chemicals, Hawthorn, Australia) was recrystallized from methanol prior to use. 1,4-Dioxane (NMR external standard) was purchased from Acros Organics (Thermo Fisher Scientific, Scoresby, Australia) and used as received. DMSO-*d_6_* was purchased from Cambridge Laboratories (Hawthorn, Australia). Acetonitrile, methanol and diethyl ether (VWR Chemicals, Bio-Strategy Pty Limited, Melbourne, Australia) were of analytical grade and used as received. 

### 3.2. Synthesis of Molecularly Imprinted Polymers

Various amounts of initiator, functional monomer, cross-linker, and templates were used for the synthesis of CAF and THP imprinted polymers in acetonitrile (see ESI Table S2). Total monomer (fM and X) was maintained at 0.500 mmol in 5.00 mL porogen, giving a 10 mL/mmol solvent to monomer ratio. After purging with nitrogen gas for 15 min, the reaction mixture was polymerised for 24 h in a water bath (F12-ED Refrigerated/Heating Circulator, Julabo, John Morris Group, Sydney, Australia) at 60 °C. The microspheres were separated from the post-polymerisation solution by centrifugation for 20 min at 2500 rpm and the post-polymerisation centrifugates stored for NMR analyses. 

Subsequently, the template was extracted by stirring the collected microspheres with approximately 3 mL of methanol:acetic acid solution (90:10 by volume) overnight and washing 3× with 3 mL methanol then a further 1 mL of methanol for ^1^H-NMR analysis to check if template extraction has been exhaustive. This extraction procedure was repeated until no template was detected in the centrifugate by ^1^H-NMR. The microspheres were then washed with diethyl ether and placed in a vacuum oven at 40 °C for further drying. 

### 3.3. Determination of Polymer Composition and Template Incorporation

Polymer composition and template incorporation were determined by calculating the amounts of left-over monomers and template in solution, post-polymerisation, by quantitative ^1^H-NMR (q-NMR) on a 600-MHz Avance III instrument (Bruker, Billerica, MA, USA). Filtered centrifugate (500 µL) was placed in a 5-mm probe while 500 µL 1,4-dioxane, STD (the reference standard) in DMSO-*d_6_* was enclosed in a coaxial insert. Spectra of both the initial and the post polymerisation solutions were acquired and processed using Bruker Topspin 3.2 software. Calibration curves were prepared using the following peaks: O-C**H**_2_ signal at 4.32 ppm for EGDMA, N-C**H**_3_ signal at 4.23 ppm for CAF (CAF-**H**1) and N-C**H** signal at 3.84 ppm for THP (THP-**H**1). The calibration curve for MAA was derived by subtracting the O-C**H**_2_ EGDMA peak from the C**H**_2_=C- signals for MAA and EGDMA at 5.10 and 5.65 ppm. These peaks are labelled in Figure 7 and were chosen because they do not overlap with the acetonitrile solvent peak at ~2.7 ppm ensuring flat baseline and accurate integration. An example of a ^1^H-NMR spectrum of the polymerisation solution showing the peaks monitored for q-NMR is given in ESI Figure S9.

### 3.4. Template Rebinding Studies

For template rebinding experiments, 10.0 mg polymers were incubated in 1.00 mL of 100 μM rebinding solution in acetonitrile for 18 h [46]. The post-rebinding solution was collected by centrifugation and the amount of template left in the solution was quantified using a LC-20AD HPLC instrument (Shimadzu, Kyoto, Japan) equipped with an Econosphere^TM^ C18, 5 μm column (Grace, Sydney, Australia), LC-20 AD pump, an SPD-20A UV detector and SIL-20A/20AC injector operated with SIL-20A autosampler. Using two solvent gradient elution consisting of 25% of a mixture of acetonitrile: water 70:30 with 10 mM trimethylamine and 75% of a 50 mM aqueous phosphate buffer (pH = 3.5) with a run time of 10 min at a flow rate of 1.0 mL/min and detection wavelength of 270 nM. A calibration curve was generated for every batch binding analyses conducted using 7 standard solutions of CAF and THP in the range of 10–1000 μM.

The HPLC rebinding results were verified by q-NMR by following the rebinding procedure above. Selected post rebinding solutions were collected and subjected to NMR analyses using 10 µM 1,4-dioxane internal standard and monitoring the same peaks for CAF (4.23, CAF-**H**1) and THP (3.84 ppm, THP-**H**1) as those used in the determination of polymer composition. 

### 3.5. Binding Selectivity Studies

The affinity of the MIPs and NIPs towards the two templates was tested by incubating 10.0 mg of the polymers in a mixture of 0.50 mL of 1.0 µM solution of CAF and 0.50 mL of 1.0 µM THP and shaken for 18 h. The suspensions were centrifuged and the centrifugates were subjected to q-^1^H NMR analyses using 10 µM 1,4-dioxane internal standard. 

### 3.6. Sample Morphology

Scanning electron microscopy (SEM) imaging was conducted using a SEM Gemini instrument (ZEISS, Oberkochen, Germany). Dried microspheres were gold coated thrice using an SPI- Module sputter coater (SPI Supplies, West Chester, PA, USA): twice in 45-degree angle and once lying flat, prior to SEM imaging. Images of the particles were obtained using a magnification of 10,000–30,000 and were analysed using the Zeiss Zen lite 2012 software (Version 2012, ZEISS, Oberkochen, Germany).

### 3.7. Particle Size Analyses

Dynamic light scattering (DLS) measurements were carried out using a Malvern Zetasizer Nano ZS with DTS Version 5.03 a software package (Malvern Instruments Ltd., Worcestershire, UK). Approximately 0.1 mg of the sample was suspended in ~0.5 mL of acetonitrile and sonicated using a benchtop ultrasonicator for ten seconds to minimise aggregation of particles. Three measurements were carried out for each sample and average sizes are expressed in terms of intensity weighted size distributions based on hydrodynamic diameters (*d*_H_). 

## 4. Conclusions

In this study, we investigated the effect of feed formulation (T:fM and fM:X ratios) and initiator concentration (i.e., I:tM ratio) on the binding performance of CAF and THP imprinted microspheres obtained by precipitation polymerisation of MAA and EGDMA as fM and X, respectively, in acetonitrile (10 mL/mmol total monomer) at 60 °C. Additionally, template incorporation and polymer conversion have been successfully monitored by quantitative ^1^H-NMR spectroscopy. 

Template incorporation ranged from 8 to 77% and, in all cases, higher for THP than CAF. It was observed to be more favourable at both high (1:2, TM2) and low (1:8, TM8), T:fM ratios, low fM:X ratios and high initiator concentration (IM5). With the exception of TM2, the T:fM ratio for all MIPs were calculated to be lower than their feed ratios, a consequence of template dilution at high solvent volume. Template binding for MIPs prepared from 1:2 to 1:8 T:fM ratios were comparable and not correlated to the level of template incorporation which suggest that, for the systems under study, the imprinting efficiency is dependent on the formation of strongly associated T-fM complexes and not on the amount of template or functional monomer in the feed. While the MIP microspheres synthesized from higher fM and lower X feed content (MX2) gave the highest template incorporation and binding capacities for both CAF and THP, their corresponding NIPs also showed high binding that resulted to lower IFs. The MX10 MIPs (low fM and high X content), on the other hand, incorporated and bound the lowest amounts of template but exhibited the most favourable binding performance, i.e., high IFs, attributed to low NIP binding. These results confirmed the importance of rigidity of the MIPs to their overall performance. This study also demonstrated the influence of the rate of polymerisation on the binding performance of MIPs, with MIPs prepared with high initiator concentration, i.e., I:tM ratio of 1:5, giving the highest IFs. The binding capacities for MIPs were less than 10% of the measured incorporated template with the exception of MX2_CAF_ (25%).

The NIPs showed no preference for either CAF or THP in competitive selectivity studies while MIPs were observed to bind preferentially to their template with THP displaying higher selectivity (72–94%) than CAF (63–84%). MIP selectivity has been observed to be significantly affected by initiator concentration with MIPs prepared with high concentration of initiator, i.e., I:tM ratio of 1:5, shown to be the most selective, i.e., 84% and 93% for IM5_CAF_ and IM5_THP_, respectively. The fM:X ratio only showed minimal effect on MIP selectivity.

We have demonstrated that, for the MIP systems under study, template incorporation, binding capacity, imprinting factor and selectivity are enhanced at a faster rate of polymerisation and thus the I:tM ratio should be maintained at (or higher than) 1:5.

The particle sizes of the microspheres were shown to be influenced by the feed formulation and concentration of initiator and are between 66 to 140 nm. Within the MIP series prepared from various T:fM ratios, TM2, containing the highest amount of template in the feed, produced the smallest particles. The MIPs of the MX series were also significantly smaller than their NIP counterparts and particle size was observed to decrease with increasing concentration of MAA from MX2 to MX10. These results indicate the ability of the template (by virtue of its interaction with the fM) to act as a nucleation point during the polymerisation process resulting in smaller particles. The particle size of IM MIPs was found to increase with increasing initiator concentration. Their NIPs, however, didn’t follow the same trend and, aside from being more polydispersed, those prepared with the highest (IM5) and lowest (IM1000), initiator concentration gave bigger particles that those with moderate initiator concentration.

It is worth noting that the results we have presented herein may be unique to the CAF and THP imprinted systems prepared under the conditions outlined in this study and may not be true for other precipitation MIP systems. Certainly, while the conditions used for this study, particularly the porogen/monomer ratio, i.e., 10 mL per mmol of total monomer, is within the range that has been employed for the synthesis of other MIPs (different template, functional monomer, crosslinker or porogen) by precipitation polymerisation (e.g., [9,48,49,50]), our results may not be comparable to other CAF and THP MIP systems that have been prepared at different porogen/monomer ratios despite employing similar initiator, functional monomer, crosslinker and porogen. Nevertheless, the results presented herein give further insight on molecular imprinting by precipitation polymerisation under dilute solution conditions. 

## Figures and Tables

**Table 1:** Binding selectivity assays for CAF and THP MIPs and NIPs.

Polymers	Template	Single Analyte Binding		Competitive Binding			
		CAF (µmol)	THP (µmol)	CAF		THP	
				µmol	f_B_^1^	µmol	f_B_^1^
**IM1000**	CAF	3.61 ± 0.10	-	1.93 ± 0.07	0.63	1.12 ± 0.13	0.37
	THP	-	4.59 ± 0.21	0.97 ± 0.02	0.28	2.52 ± 0.20	0.72
	NIP	3.32 ± 0.10	3.86 ± 0.28	1.50 ± 0.03	0.52	1.36 ± 0.04	0.48
**IM100 ^2^** **MX5** **TM4**	CAF	4.70 ± 0.20	-	3.76 ± 0.01	0.77	1.15 ± 0.07	0.23
	THP	-	5.40 ± 0.20	0.79 ± 0.12	0.13	5.92 ± 0.10	0.88
	NIP	3.59 ± 0.15	3.55 ± 0.29	1.33 ± 0.15	0.58	0.96 ± 0.15	0.42
**IM5**	CAF	4.91 ± 0.24	-	4.52 ± 0.25	0.84	0.87 ± 0.04	0.16
	THP	-	5.97 ± 0.36	0.40 ± 0.01	0.07	5.38 ± 0.02	0.93
	NIP	2.82 ± 0.12	3.07 ± 0.29	1.38 ± 0.03	0.51	1.32 ± 0.01	0.49
**MX2**	CAF	14.10 ± 0.33	-	9.64 ± 0.05	0.69	4.31 ± 0.01	0.31
	THP	-	14.7 ± 1.10	1.36 ± 0.01	0.11	11.83 ± 0.61	0.89
	NIP	12.25 ± 0.07	11.97 ± 1.21	5.28 ± 0.05	0.47	6.01 ± 0.02	0.53
**MX10**	CAF	3.80 ± 0.01	-	2.28 ± 0.06	0.73	0.85 ± 0.01	0.27
	THP	-	4.00 ± 0.20	0.23 ± 0.01	0.06	3.75 ± 0.04	0.94
	NIP	2.36 ± 0.01	2.12 ± 0.15	1.40 ± 0.13	0.59	0.98 ± 0.11	0.41

^1^ Binding with respect to total CAF + THP binding. ^2^ This polymer has been used for the I:tM, fM:X and T:fM studies and thus has been assigned 3 different codes.

## Supplementary Materials

Supplementary Materials are available online. Table S1: Summary of imprinting results, Table S2: Polymer feed compositions, Figure S1: SEM images of microspheres from various T:fM ratios, Figure S2: Particle sizes of microspheres from various T:fM ratios, Figure S3: Incorporation of CAF and THP in polymers from various T:fM ratios, Figure S4: Incorporation of CAF and THP in polymers from various fM:X ratios, Figure S5: CAF incorporation and rebinding by MIPs and NIPs from various fM:X ratios, Figure S6: THP incorporation and rebinding by MIPs and NIPs from various fM:X ratios, Figure S7: SEM images of microspheres from various I:tM ratios, Figure S8: CAF and THP incorporation in polymers from various I:tM ratios, Figure S9: An example of a 1H NMR spectrum of the caffeine pre-polymerization solution.
