# Supplementary material for: Effect of Formulation on the Binding Efficiency and Selectivity of Precipitation Molecularly Imprinted Polymers
Source: Molecules. 2018 Nov 16;23(11):2996. doi: 10.3390/molecules23112996 (PMC6278369; doi:10.3390/molecules23112996)
Supplement: Supplementary file 1 [file molecules-23-02996-s001.pdf]

Effect of Formulation on the Binding Efficiency and Selectivity of Precipitation MIPs

K. Fremielle Lim and Clovia I. Holdsworth

Discipline of Chemistry, School of Environmental and Life Sciences, University of Newcastle, Callaghan, NSW 2308 Australia

Fax: +612 49215472; Tel: +612 9215481; E-mail: Clovia.Holdsworth@newcastle.edu.au

Electronic Supplementary Information

Table S1. Imprinting results of polymers synthesized from various template:functional monomer (TM), functional monomer:cross-linker (MX) and initiator:total monomer (IM) ratios.

Experiments		Incorporated components in the polymers % incorporation (µmol)			Polymer Composition T :FM : XL[1]	Degree of Cross-linking[2]	Hydro-dynamic size, d_H (PDI)
		EGDMA	MAA	Template			
**TM2**	MIP_{CAF}	90 ± 1 (375)	79 ± 1 (66)	70 ± 1 (29)	0.44:1:6	68.1 ± 0.01	66 ± 1 (1.0)
	MIP_{THP}	92 ± 1 (382)	75 ± 1 (63)	76 ± 1 (32)	0.51:1:6	62.3 ± 0.01	70 ± 1 (1.0)
	NIP	83 ± 7 (347)	75 ± 2 (62)		1:5.58	70.3 ± 0.06	104 ± 1 (0.4)
**TM4/ MX5/ IM100[3]**	MIP_{CAF}	89 ± 2 (369)	80 ± 2 (67)	18 ± 1 (4)	0.06:1:5.53	71.0 ± 0.01	114 (0.3)
	MIP_{THP}	85 ± 2 (359)	77 ± 1 (64)	49 ± 2 (10)	0.16:1:5.10	69.7 ± 0.01	100 ± 1 (0.3)
	NIP	92 ± 2 (384)	80 ± 1 (67)		1:5.77	71.0 ± 0.01	93 ±)1 (1.0)
**TM6**	MIP_{CAF}	87 ± 2 (362)	74 ± 3 (62)	35 ± 1 (5)	0.08:1:5.85	63.3 ± 0.01	99 ± 2 (0.)
	MIP_{THP}	90 ± 2 (373)	77 ± 2 (64)	49 ± 1 (7)	0.11:1:5.82	60.6 ± 0.01	85 ± 1 (0.5)
	NIP	92 ± 2 (382)	88 ± 4 (73)		1:5.24	63.3 ± 0.01	93 ± 1 (0.2)
**TM8**	MIP_{CAF}	93 ± 1 (387)	87 ± 1 (72)	64 ± 1 (7)	0.09:1:5.36	62.3 ± 0.01	75 ± 1 (0.8)
	MIP_{THP}	93 ± 2 (386)	82 ± 1 (69)	77 ± 1 (8)	0.12:1:5.63	61.0 ± 0.01	98 ± 2 (0.3)
	NIP	86 ± 2 (360)	79 ± 1 (65)		1:5.57	68.7 ± 0.01	89 ± 1 (0.5)
**MX10**	MIP_{CAF}	88 ± 1 (409)	70 ± 1 (32)	35 ± 1 (4)	0.08:1:12.60	63.8 ± 0.1	117 ± 1 (0.2)
	MIP_{THP}	86 ± 2 (392)	61 ± 1 (28)	32 ± 1 (4)	0.13:1:14.15	75.7 ± 0.1	114 ± 1 (0.2)
	NIP	86 ± 2 (390)	79 ± 2 (36)		1:10.89	63.3 ± 0.1	128 ± 1 (0.5)

[1]T:fM:X = template : functional monomer : cross-linker (mol) ratio in the polymers, [2]Degree of cross-linking compared to the initial ratio -C=C- and -C=O- and in the polymers. [3] Polymers produced using T:fM = 1:4, fM:X = 1:5 and I:tM of 1:100.

Table S1 (continuation). Imprinting results of polymers synthesized from various template:functional monomer (TM), functional monomer:cross-linker (MX) and initiator:total monomer (IM) ratios.

Experiments		Incorporated components in the polymers % incorporation (µmol)			Polymer Composition T :FM : XL[1]	Degree of Cross-linking[2]	Hydro-dynamic size, d_H (PDI)
		EGDMA	MAA	Template			
**MX2**	MIP_{CAF}	93 ± 2 (311)	78 ± 2 (130)	10 ± 2 (4)	0.03:1:2.41	49.6± 0.1	84 ± 1 (0.1)
	MIP_{THP}	93 ± 2 (310)	83 ± 2 (138)	31 ± 0 (13)	0.09:1:2.26	57.7 ± 0.1	67 ± 1 (0.1)
	NIP	93 ± 2 (366)	84 ± 1 (57)		1:2.21	59.1 ± 0.1	89 ± 1 (0.5)
**IM5**	MIP_{CAF}	98 ± 1 (410)	92 ± 2 (77)	26 ± 1 (12)	0.07:1:5.33	67.5 ± 0.01	136 ± 1 (0.2)
	MIP_{THP}	98 ± 1 (410)	93 ± 2 (77)	50 ± 1 (10)	0.13:1:5.33	68.4 ± 0.01	132 ± 1 (0.2)
	NIP	98 ± 1 (409)	88 ± 2 (73)		1:5.58	73.6 ± 0.06	122 ± 1 (0.3)
**IM10**	MIP_{CAF}	96 ± 1 (401)	84 ± 2 (70)	21 ± 1 (4)	0.06:1:5.75	72.2 ± 0.01	121 ± 1 (0.6)
	MIP_{THP}	96 ± 0 (398)	82 ± 1 (69)	52 ± 3 (11)	0.16:1:5.81	73.1 ± 0.01	102 ± 1 (0.6)
	NIP	97 ± 1 (404)	83 ± 1 (69)		1:5.83	69.4 ± 0.01	83 ± 2 (1.0)
**IM500**	MIP_{CAF}	64 ± 2 (266)	56 ± 0 (47)	16 ± 1 (3)	0.10:1:5.37	67.3 ± 0.01	104 ± 1 (0.3)
	MIP_{THP}	61 ± 2 (256)	57 ± 2 (48)	22 ± 0 (5)	0.07:1:5.69	65.2 ± 0.01	94 ± 1 (0.3)
	NIP	88 ± 2 (366)	69 ± 1 (57)		1:6.41	67.3 ± 0.01	140 ± 1 (0.3)
**IM1000**	MIP_{CAF}	40 ± 0 (165)	39 ± 2 (32)	8 ± 1 (2)	0.05:1:5.16	66.6 ± 0.01	107 ± 1 (0.3)
	MIP_{THP}	43 ± 2 (179)	38 ± 2 (32)	12 ± 1 (3)	0.08:1:5.64	67.4 ± 0.01	90 ± 1 (0.3)
	NIP	75 ± 3 (314)	39 ± 2 (32)		1:9.70	62.3 ± 0.01	119 ± 1 (0.3)

[1]T:fM:X = template : functional monomer : cross-linker (mol) ratio in the polymers, [2]Degree of cross-linking compared to the initial ratio -C=C- and -C=O- and in the polymers. [3]Polymers produced using T:fM = 1:4, fM:X = 1:5 and I:tM of 1:100.

Table S2. Polymer feed composition of the systems investigated from various template:functional monomer (TM), functional monomer:cross-linker (MX) and initiator:total monomer (IM) ratios.

<table>
<tr><th>EXPT/Polymers</th><th>[template] (μmol)</th><th>[FM] (μmol)</th><th>[XL] (μmol)</th><th>[initiator] (μmol)</th><th>Volume of porogen (mL)</th><th>T:FM:XL</th><th>I:TM</th></tr>
<tr><td>IM1000</td><td rowspan="5">20.80</td><td rowspan="5">83.33</td><td rowspan="5">416.67</td><td>0.50</td><td rowspan="10">5.00</td><td rowspan="5">1:4:20</td><td>1:1000</td></tr>
<tr><td>IM500</td><td>1.0</td><td>1:500</td></tr>
<tr><td>IM100, MX5, TM4</td><td>5.00</td><td>1:100</td></tr>
<tr><td>IM10</td><td>50.00</td><td>1:10</td></tr>
<tr><td>IM5</td><td>100.00</td><td>1:5</td></tr>
<tr><td>MX2</td><td>42.00</td><td>167.00</td><td>333.00</td><td rowspan="5">5.00</td><td>1:4:8</td><td rowspan="5">1:100</td></tr>
<tr><td>MX10</td><td>14.00</td><td>45.00</td><td>455.00</td><td>1:40:40</td></tr>
<tr><td>TM2</td><td>42.00</td><td rowspan="3">83.00</td><td rowspan="3">416.67</td><td>1:2:10</td></tr>
<tr><td>TM6</td><td>21.00</td><td>1:4:20</td></tr>
<tr><td>TM8</td><td>10.00</td><td>1:8:40</td></tr>
</table>

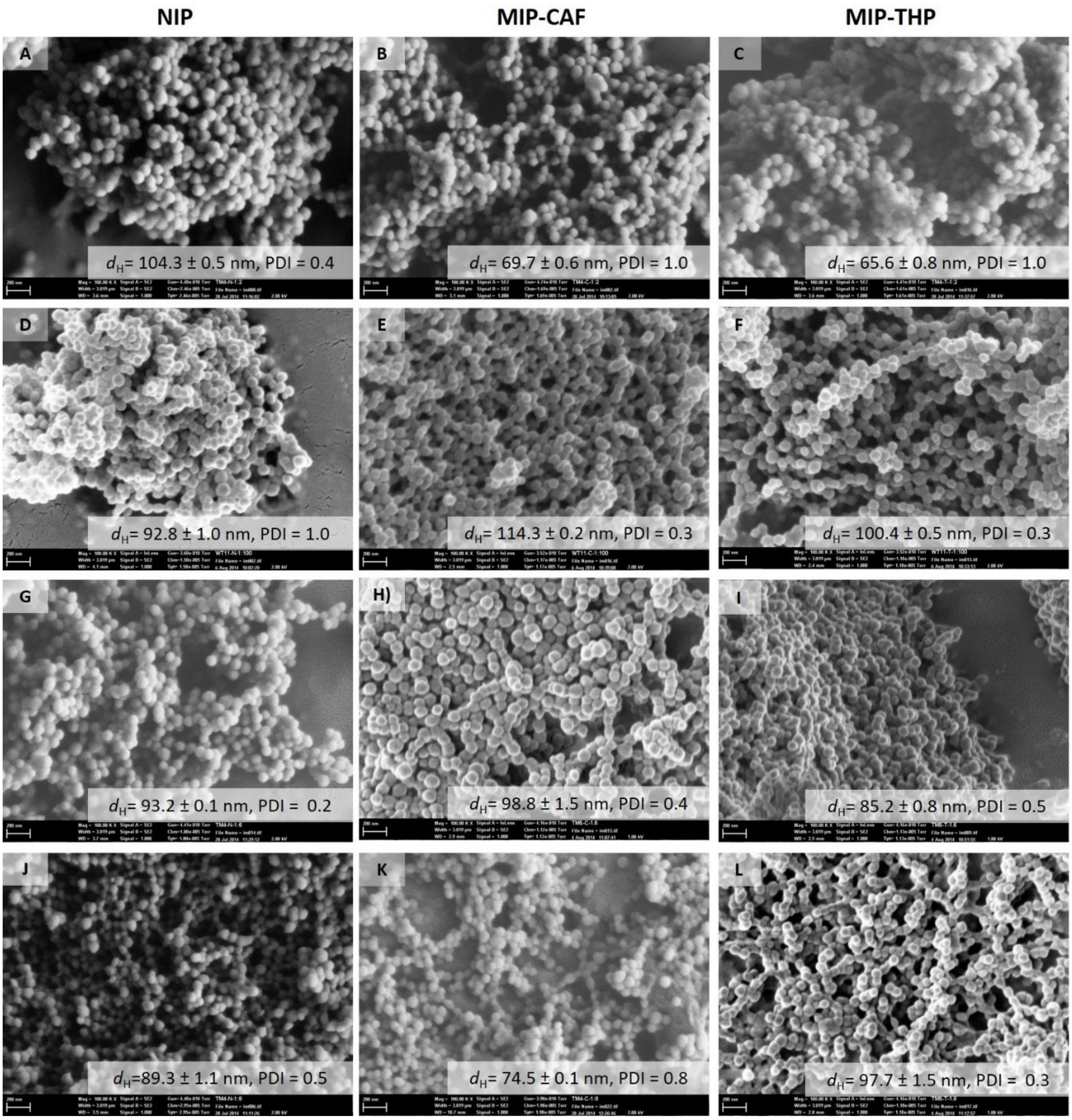

Figure S1. SEM images of microspheres synthesized from various template:functional monomer (T:fM) ratios: (**A**) TM2$_{NIP}$, (**B**) TM2$_{CAF}$, (**C**) TM2$_{THP}$, (**D**) TM4$_{NIP}$, (**E**) TM4$_{CAF}$, (**F**) TM4$_{THP}$, (**G**) TM6$_{NIP}$, (**H**) TM6$_{CAF}$, (**I**) TM6$_{THP}$, (**J)** TM8$_{NIP}$, (**K**) TM8$_{CAF}$ and (**L**) TM8$_{THP}$. Scale bar represents 200 μm at 100000x magnification. Insets are the hydrodynamic size of the microspheres with the corresponding polydispersity indexes (PDI) measured by DLS.

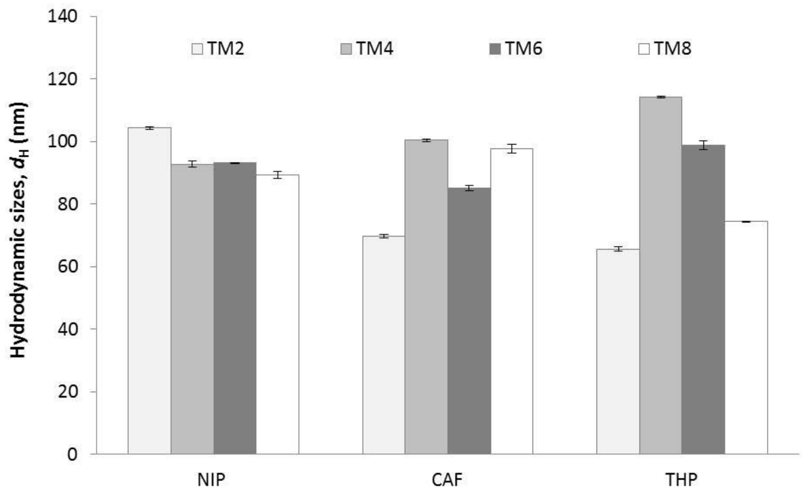

Figure S2. Hydrodynamic sizes, d_H, of microspheres synthesized from various T:fM ratios. Measurements were conducted using Dynamic Light scattering (DLS) and acetonitrile as the dispersant.

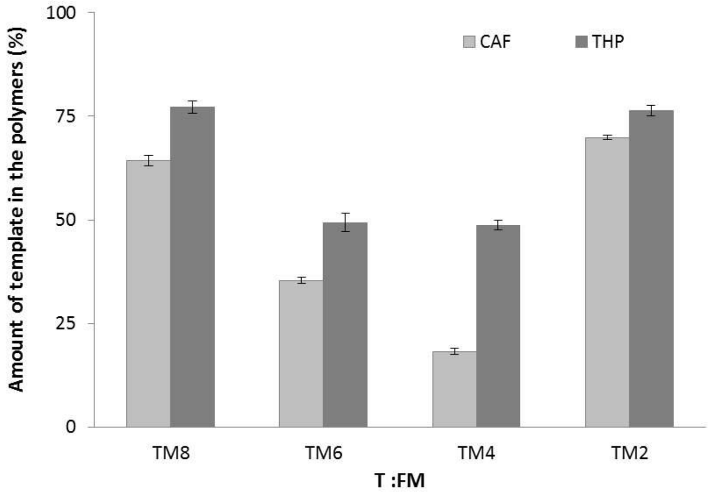

Figure S3. CAF and THP incorporated in the polymers from various T:fM ratios measured by ^{1}H NMR using 1,4-dioxane in DMSO-d_6 as the reference standard.

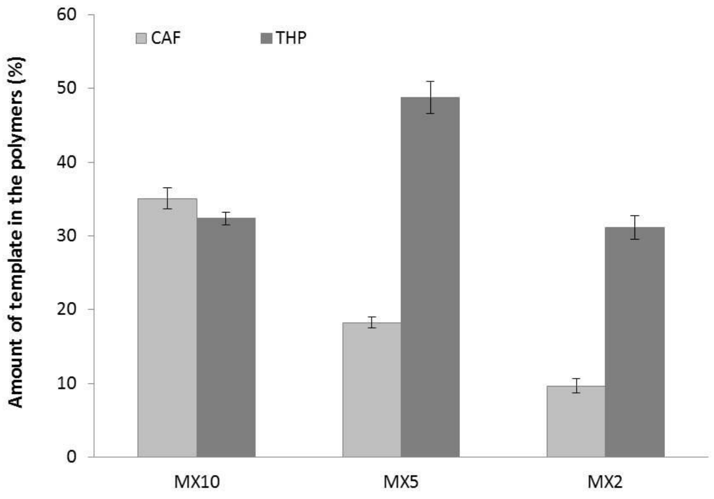

Figure S4. CAF and THP incorporated in the polymers from various fM:X ratios. Measured by ^{1}H NMR using 1,4-dioxane in DMSO-d_6 as the reference standard.

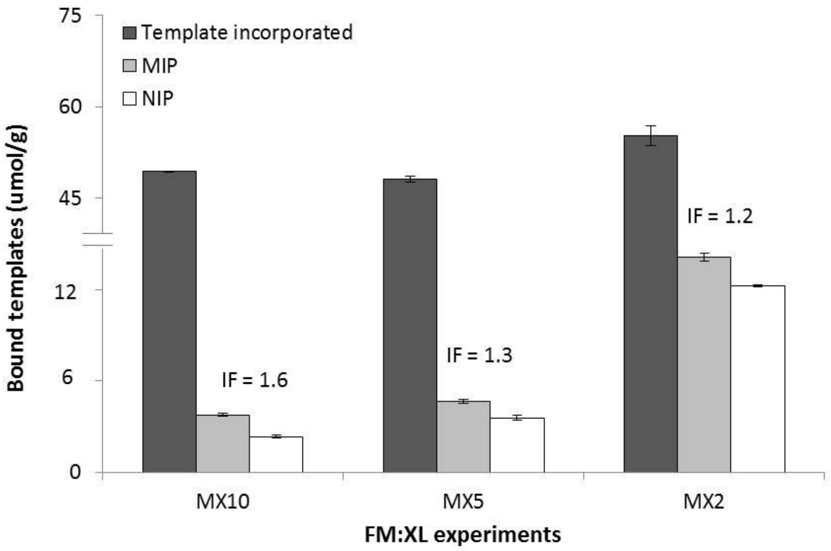

Figure S5. CAF incorporation and rebinding of MIPs and NIPs prepared from various fM:X ratios. Polymers were incubated for 18 hours in 100µM template rebinding solution and the post rebinding solutions were analyzed by HPLC.

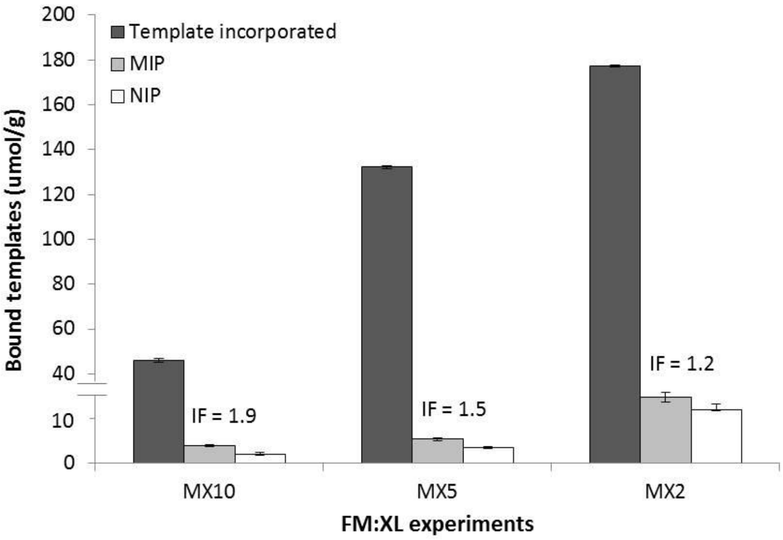

Figure S6. THP incorporation and rebinding of MIPs and NIPs prepared from various fM:X ratios. Polymers were incubated for 18 hours in 100µM template rebinding solution and the post rebinding solutions were analysed by HPLC.

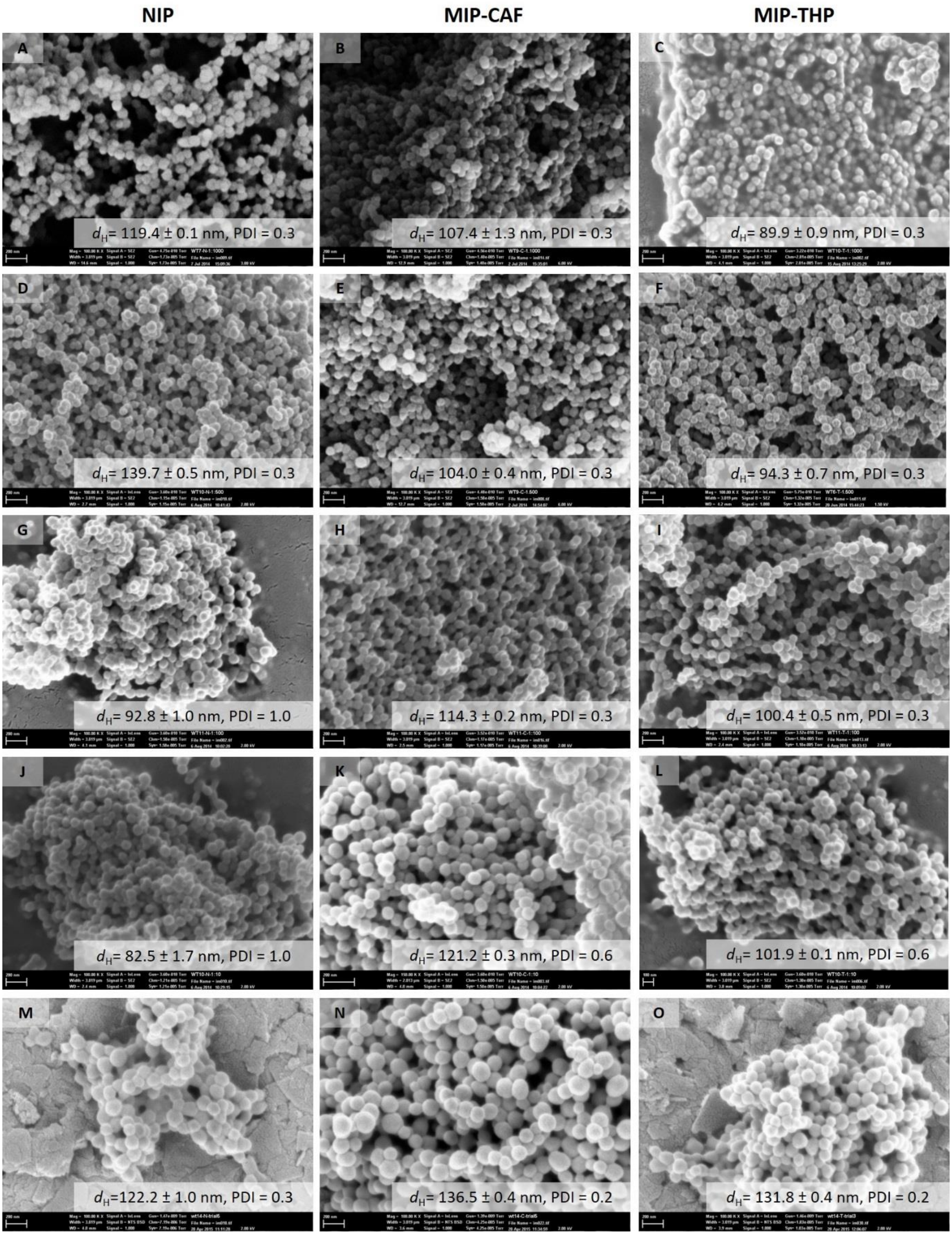

Figure S7. SEM images of microspheres synthesized from various initiator:total monomer (I:tM) ratios: (**A**) $IM1000_{NIP}$, (**B**) $IM1000_{CAF}$, (**C**) $IM1000_{THP}$, (**D**) $IM500_{NIP}$, (**E**) $IM500_{CAF}$, (**F**) $IM500_{THP}$, (**G**) $IM100_{NIP}$, (**H**) $IM100_{CAF}$, (**I**) $IM100_{THP}$, (**J**) $IM10_{NIP}$, (**K**) $IM10_{CAF}$, (**L**) $IM10_{THP}$, (**M**) $IM5_{NIP}$, (**N**) $IM5_{CAF}$ and (**O**) $IM5_{THP}$. Scale bar represents 200 μm at 100000x magnification. Insets are the hydrodynamic size of the microspheres with the corresponding polydispersity indexes (PDI) measured by DLS.

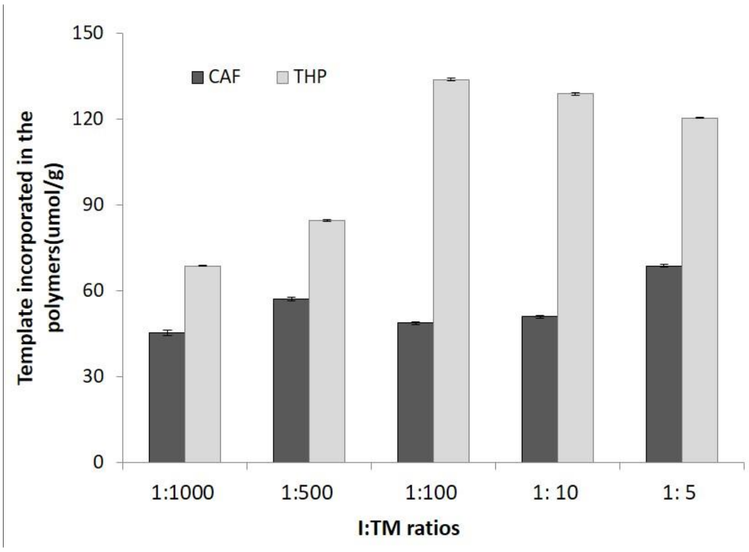

Figure S8. CAF and THP incorporation the polymers from various I:tM ratios measured by ^{1}H NMR using 1,4-dioxane in DMSO-d_6 as the reference standard.

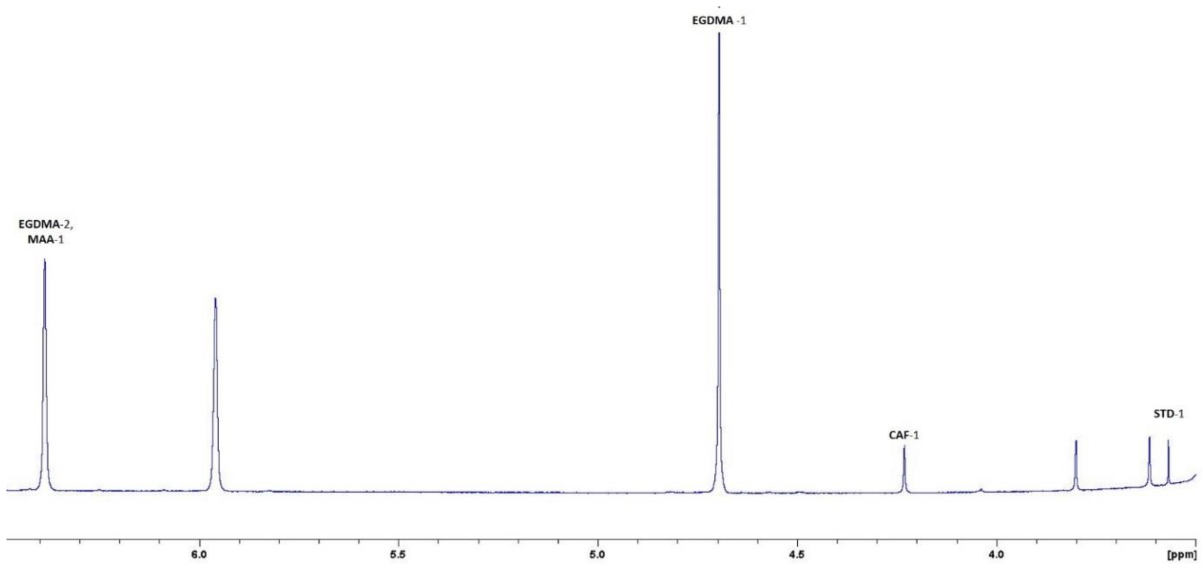

Figure S9. An example of a ^{1}H NMR spectrum of the caffeine pre-polymerization solution. Peaks of the components are as follows; cross-linker (EGDMA, O-C**H**$_2$ signal) at 4.32 ppm, functional monomer (the combination of the signals of -CH_2=C**H**$_2$- of MAA and EGDMA) at 5.96 and 6.39 ppm and for caffeine at 4.23 ppm (-N-C**H**$_3$).
